# Supplementary material for: A UMLS-based spell checker for natural language processing in vaccine safety
Source: BMC Med Inform Decis Mak. 2007 Feb 12;7:3. doi: 10.1186/1472-6947-7-3 (PMC1805499; doi:10.1186/1472-6947-7-3)
Supplement: Additional file 13 — RAP application directory. Sets up directory for RAP (RDF application for PHP) source code [file 1472-6947-7-3-S13.gz › rap/doc/phpdoc/elementindex_dataset.html]

xml version="1.0" encoding="iso-8859-1"?


## [dataset] element index

### Package indexes

- model
- infModel
- resModel
- ontModel
- syntax
- rdql
- utility

All elements
  

a
c
d
e
f
g
h
i
k
l
n
p
q
r
s
t
v

a

top

addAll
:   DatasetMem::addAll() in DatasetMem.php

    Add all named graphs of the other dataset to this dataset

addAll
:   DatasetDb::addAll() in DatasetDb.php

    Add all named graphs of the other dataset to this dataset

addNamedGraph
:   DatasetMem::addNamedGraph() in DatasetMem.php

    Adds a NamedGraph to the set. Will replace a NamedGraph with the same name that is already in the set.

addNamedGraph
:   DatasetDb::addNamedGraph() in DatasetDb.php

    Adds a NamedGraph to the set.

addQuad
:   DatasetMem::addQuad() in DatasetMem.php

    Adds a quad to the Dataset. The argument must not contain any wildcards. If the quad is already present, nothing happens. A new named graph will automatically be created if necessary.

addQuad
:   DatasetDb::addQuad() in DatasetDb.php

    Adds a quad to the Dataset. The argument must not contain any wildcards. If the quad is already present, nothing happens. A new named graph will automatically be created if necessary.

c

top

clear
:   DatasetDb::clear() in DatasetDb.php

    Deletes all NamedGraphs from the set.

clear
:   DatasetMem::clear() in DatasetMem.php

    Deletes all NamedGraphs from the set.

containsNamedGraph
:   DatasetMem::containsNamedGraph() in DatasetMem.php

    Tells wether the Dataset contains a NamedGraph.

containsNamedGraph
:   DatasetDb::containsNamedGraph() in DatasetDb.php

    Tells wether the Dataset contains a NamedGraph.

containsQuad
:   DatasetMem::containsQuad() in DatasetMem.php

    Tells wether the Dataset contains a quad or quads matching a pattern.

containsQuad
:   DatasetDb::containsQuad() in DatasetDb.php

    Tells wether the Dataset contains a quad or quads matching a pattern.

countGraphs
:   DatasetDb::countGraphs() in DatasetDb.php

    Returns the number of NamedGraphs in the set. Empty graphs are counted.

countGraphs
:   DatasetMem::countGraphs() in DatasetMem.php

    Returns the number of NamedGraphs in the set. Empty graphs are counted.

countQuads
:   DatasetMem::countQuads() in DatasetMem.php

    Counts the Quads in the RDF dataset. Identical Triples in different NamedGraphs are counted individually.

countQuads
:   DatasetDb::countQuads() in DatasetDb.php

    Counts the Quads in the RDF dataset. Identical Triples in different NamedGraphs are counted individually.

createGraph
:   DatasetDb::createGraph() in DatasetDb.php

    Creates a new NamedGraph and adds it to the set. An existing graph with the same name will be replaced. But the old namedGraph remains in the database.

createGraph
:   DatasetMem::createGraph() in DatasetMem.php

    Creates a new NamedGraph and adds it to the set. An existing

current
:   IteratorFindQuadsMem::current() in IteratorFindQuadsMem.php

    Returns the current item

current
:   IteratorFindQuadsDb::current() in IteratorFindQuadsDb.php

    Returns the current item

current
:   IteratorAllGraphsMem::current() in IteratorAllGraphsMem.php

    Returns the current item

current
:   IteratorAllGraphsDb::current() in IteratorAllGraphsDb.php

    Returns the current item

d

top

Dataset
:   Dataset in Dataset.php

    Dataset implementation.

DatasetDb
:   DatasetDb::DatasetDb() in DatasetDb.php

    Constructor You can supply a Dataset name.

DatasetDb
:   DatasetDb in DatasetDb.php

    Dataset implementation.

DatasetMem
:   DatasetMem in DatasetMem.php

    In-memory implementation of a RDF dataset.

DatasetMem
:   DatasetMem::DatasetMem() in DatasetMem.php

    Constructor You can supply a Dataset name.

DatasetDb.php
:   DatasetDb.php in DatasetDb.php

DatasetMem.php
:   DatasetMem.php in DatasetMem.php

Dataset.php
:   Dataset.php in Dataset.php

e

top

equals
:   Quad::equals() in Quad.php

    Checks, if two quads are equal.

f

top

findInDefaultGraph
:   DatasetMem::findInDefaultGraph() in DatasetMem.php

    Finds Statements that match a pattern in the default Graph. The argument may contain wildcards.

findInDefaultGraph
:   DatasetDb::findInDefaultGraph() in DatasetDb.php

    Finds Statements that match a pattern in the default Graph. The argument may contain wildcards.

findInNamedGraphs
:   DatasetMem::findInNamedGraphs() in DatasetMem.php

    Finds Statements that match a quad pattern. The argument may contain wildcards.

findInNamedGraphs
:   DatasetDb::findInNamedGraphs() in DatasetDb.php

    Finds Statements that match a quad pattern. The argument may contain wildcards.

g

top

getDatasetName
:   DatasetMem::getDatasetName() in DatasetMem.php

    Return the Dataset name.

getDatasetName
:   DatasetDb::getDatasetName() in DatasetDb.php

    Return the Dataset name.

getDefaultGraph
:   DatasetMem::getDefaultGraph() in DatasetMem.php

    Returns a reference to the defaultGraph

getDefaultGraph
:   DatasetDb::getDefaultGraph() in DatasetDb.php

    Returns a reference to the defaultGraph

getGraphName
:   NamedGraphMem::getGraphName() in NamedGraphMem.php

    Return the graph name.

getGraphName
:   Quad::getGraphName() in Quad.php

    Return the graph name.

getGraphName
:   NamedGraphDb::getGraphName() in NamedGraphDb.php

    Return the graph name.

getNamedGraph
:   DatasetDb::getNamedGraph() in DatasetDb.php

    Returns the NamedGraph with a specific name from the Dataset.

getNamedGraph
:   DatasetMem::getNamedGraph() in DatasetMem.php

    Returns the NamedGraph with a specific name from the Dataset.

getObject
:   Quad::getObject() in Quad.php

    Return the object.

getPredicate
:   Quad::getPredicate() in Quad.php

    Return the predicate.

getStatement
:   Quad::getStatement() in Quad.php

    Return the statement(subject,predicate,object).

getSubject
:   Quad::getSubject() in Quad.php

    Return the subject.

h

top

hasDefaultGraph
:   DatasetMem::hasDefaultGraph() in DatasetMem.php

    Returns true, if an defaultGraph exists. False otherwise

hasDefaultGraph
:   DatasetDb::hasDefaultGraph() in DatasetDb.php

    Returns true, if an defaultGraph exists. False otherwise

i

top

IteratorAllGraphsDb.php
:   IteratorAllGraphsDb.php in IteratorAllGraphsDb.php

IteratorAllGraphsMem.php
:   IteratorAllGraphsMem.php in IteratorAllGraphsMem.php

IteratorFindQuadsDb.php
:   IteratorFindQuadsDb.php in IteratorFindQuadsDb.php

IteratorFindQuadsMem.php
:   IteratorFindQuadsMem.php in IteratorFindQuadsMem.php

isEmpty
:   DatasetDb::isEmpty() in DatasetDb.php

    Tells wether the set contains any NamedGraphs.

isEmpty
:   DatasetMem::isEmpty() in DatasetMem.php

    Tells wether the set contains any NamedGraphs.

IteratorAllGraphsDb
:   IteratorAllGraphsDb in IteratorAllGraphsDb.php

    Implementation of a Graph iterator.

IteratorAllGraphsDb
:   IteratorAllGraphsDb::IteratorAllGraphsDb() in IteratorAllGraphsDb.php

    Constructor.

IteratorAllGraphsMem
:   IteratorAllGraphsMem in IteratorAllGraphsMem.php

    Implementation of a Graph iterator.

IteratorAllGraphsMem
:   IteratorAllGraphsMem::IteratorAllGraphsMem() in IteratorAllGraphsMem.php

    Constructor.

IteratorFindQuadsDb
:   IteratorFindQuadsDb::IteratorFindQuadsDb() in IteratorFindQuadsDb.php

    Constructor.

IteratorFindQuadsDb
:   IteratorFindQuadsDb in IteratorFindQuadsDb.php

    Implementation of a quad iterator.

IteratorFindQuadsMem
:   IteratorFindQuadsMem::IteratorFindQuadsMem() in IteratorFindQuadsMem.php

    Constructor.

IteratorFindQuadsMem
:   IteratorFindQuadsMem in IteratorFindQuadsMem.php

    Implementation of a quad iterator.

k

top

key
:   IteratorFindQuadsMem::key() in IteratorFindQuadsMem.php

    Returns the key of the current item

key
:   IteratorFindQuadsDb::key() in IteratorFindQuadsDb.php

    Returns the key of the current item

key
:   IteratorAllGraphsMem::key() in IteratorAllGraphsMem.php

    Returns the key of the current item

key
:   IteratorAllGraphsDb::key() in IteratorAllGraphsDb.php

    Returns the key of the current item

l

top

listGraphNames
:   DatasetMem::listGraphNames() in DatasetMem.php

    Returns the names of the namedGraphs in this set as strings in an array.

listGraphNames
:   DatasetDb::listGraphNames() in DatasetDb.php

    Returns the names of the namedGraphs in this set as strings in an array.

listNamedGraphs
:   DatasetMem::listNamedGraphs() in DatasetMem.php

    Returns an iterator over all NamedGraphs in the set.

listNamedGraphs
:   DatasetDb::listNamedGraphs() in DatasetDb.php

    Returns an iterator over all NamedGraphs in the set.

loadFromFile
:   Dataset::loadFromFile() in Dataset.php

    Load a Dataset from File

loadFromString
:   Dataset::loadFromString() in Dataset.php

    Load a Datset from a string

n

top

NamedGraphDb.php
:   NamedGraphDb.php in NamedGraphDb.php

NamedGraphMem.php
:   NamedGraphMem.php in NamedGraphMem.php

NamedGraphDb
:   NamedGraphDb::NamedGraphDb() in NamedGraphDb.php

    Constructor Do not call this directly.

NamedGraphDb
:   NamedGraphDb in NamedGraphDb.php

    Persistent NamedGraph implementation that extends a DbModel.

NamedGraphMem
:   NamedGraphMem in NamedGraphMem.php

    NamedGraph implementation that extends a MemModel with a name.

NamedGraphMem
:   NamedGraphMem::NamedGraphMem() in NamedGraphMem.php

    Constructor You have to supply a graph name. You can supply a URI

next
:   IteratorFindQuadsDb::next() in IteratorFindQuadsDb.php

    Moves Iterator to the next item in the list

next
:   IteratorFindQuadsMem::next() in IteratorFindQuadsMem.php

    Moves Iterator to the next item in the list

next
:   IteratorAllGraphsMem::next() in IteratorAllGraphsMem.php

    Moves Iterator to the next item in the list

next
:   IteratorAllGraphsDb::next() in IteratorAllGraphsDb.php

    Moves Iterator to the next item in the list

p

top

parseFile
:   TriXParser::parseFile() in TriXParser.php

    Parse from a file

parseString
:   TriXParser::parseString() in TriXParser.php

    Parse an XML string

q

top

Quad.php
:   Quad.php in Quad.php

Quad
:   Quad::Quad() in Quad.php

    Constructor Creates a Quad from four Nodes.

Quad
:   Quad in Quad.php

    A Triple in a RDF dataset, consisting of four Jena Nodes: graphName, subject, predicate, and object.

r

top

removeNamedGraph
:   DatasetDb::removeNamedGraph() in DatasetDb.php

    Removes a NamedGraph from the set. Nothing happens if no graph with that name is contained in the set.

removeNamedGraph
:   DatasetMem::removeNamedGraph() in DatasetMem.php

    Removes a NamedGraph from the set. Nothing happens if no graph with that name is contained in the set.

removeQuad
:   DatasetMem::removeQuad() in DatasetMem.php

    Deletes a Quad from the RDF dataset.

removeQuad
:   DatasetDb::removeQuad() in DatasetDb.php

    Deletes a Quad from the RDF dataset.

rewind
:   IteratorFindQuadsMem::rewind() in IteratorFindQuadsMem.php

    Resets iterator list to start

rewind
:   IteratorFindQuadsDb::rewind() in IteratorFindQuadsDb.php

    Resets iterator list to start

rewind
:   IteratorAllGraphsDb::rewind() in IteratorAllGraphsDb.php

    Resets iterator list to start

rewind
:   IteratorAllGraphsMem::rewind() in IteratorAllGraphsMem.php

    Resets iterator list to start

s

top

serializeToFile
:   TriXSerializer::serializeToFile() in TriXSerializer.php

    Serialize the dataset to a TriX string and save in file

serializeToFile
:   Dataset::serializeToFile() in Dataset.php

    Serialize the Dataset to File

serializeToString
:   TriXSerializer::serializeToString() in TriXSerializer.php

    Serialize the dataset to a TriX string

serializeToString
:   Dataset::serializeToString() in Dataset.php

    Serialize the Dataset to string

setDatasetName
:   DatasetDb::setDatasetName() in DatasetDb.php

    Sets the Dataset name. Return true on success, false otherwise

setDatasetName
:   DatasetMem::setDatasetName() in DatasetMem.php

    Sets the Dataset name.

setDefaultGraph
:   DatasetMem::setDefaultGraph() in DatasetMem.php

    Overwrites the existting default graph.

setDefaultGraph
:   DatasetDb::setDefaultGraph() in DatasetDb.php

    Overwrites the existting default graph.

setGraphName
:   NamedGraphDb::setGraphName() in NamedGraphDb.php

    Sets the graph name.

setGraphName
:   Quad::setGraphName() in Quad.php

    Sets the graph name.

setGraphName
:   NamedGraphMem::setGraphName() in NamedGraphMem.php

    Sets the graph name.

t

top

TriXParser.php
:   TriXParser.php in TriXParser.php

TriXSerializer.php
:   TriXSerializer.php in TriXSerializer.php

toString
:   Quad::toString() in Quad.php

    Return a human-readable (sort of) string "graphname { s p o . }" describing the quad.

TriXParser
:   TriXParser::TriXParser() in TriXParser.php

    Constructor Needs a reference to a graphSet

TriXParser
:   TriXParser in TriXParser.php

    Temporary implementation of a TriX-Parser (Usable only with PHP > V5) Currently, it doesn't support any namespaces and has problems with typed literals.

TriXSerializer
:   TriXSerializer::TriXSerializer() in TriXSerializer.php

    Constructor Needs a reference to a graphSet

TriXSerializer
:   TriXSerializer in TriXSerializer.php

    Temporary implementation of a TriX-Serializer

v

top

valid
:   IteratorFindQuadsMem::valid() in IteratorFindQuadsMem.php

    Says if there are additional items left in the list

valid
:   IteratorFindQuadsDb::valid() in IteratorFindQuadsDb.php

    Says if there are additional items left in the list

valid
:   IteratorAllGraphsMem::valid() in IteratorAllGraphsMem.php

    Says if there are additional items left in the list

valid
:   IteratorAllGraphsDb::valid() in IteratorAllGraphsDb.php

    Says if there are additional items left in the list

a
c
d
e
f
g
h
i
k
l
n
p
q
r
s
t
v
